# Components and Outcomes in Under- and Postgraduate Medical Education to Prepare for the Delivery of Integrated Care for the Elderly: A Scoping Review

**DOI:** 10.5334/ijic.6959

**Published:** 2023-04-19

**Authors:** M. T. (Mariëlle) van Wijngaarden, D. Z. B. (Dieneke) van Asselt, S. M. (sietske) Grol, N. D. (Nynke) Scherpbier-de Haan, C. R. M. G. (Lia) Fluit

**Affiliations:** 1 Radboud University Medical Center, Radboudumc Health Academy, Research on Learning and Education, Nijmegen, the Netherlands; 2 Radboud University Medical Center, Department of Geriatric Medicine, the Netherlands; 3 Radboud University Medical Center, Corporate Staff Strategy Development, Nijmegen, the Netherlands; 4 Radboud University Medical Center, Radboud Institute for Health Sciences, Department of Primary and Community Care, Nijmegen, the Netherlands; 5 University Medical Centre Groningen, Department of General Practice and Elderly Care Medicine, Groningen, the Netherlands

**Keywords:** integrated care, comprehensive care, continuity of care, medical education, elderly

## Abstract

**Introduction:**

The ageing society requires physicians who can deliver integrated care, but it is unclear how they should be prepared for doing so. This scoping review aims to create an overview of educational programmes that prepare (future) physicians to deliver integrated care while addressing components and outcomes of the interventions.

**Method:**

We included papers from five databases that contained: (1) integrated care (2) education programme (3) medical students (4) elderly, or synonyms. We divided the WHO definition of integrated care into ten components for the concept of ‘integrated care’. Data were collected with a charting template, and template analysis was used to formulate themes.

**Results:**

We found 17 educational programmes in different learning settings. All programmes addressed several components of the WHO definition. The programmes primarily focused on care for individual patients (micro-level), and the outcomes suggested that experiencing the complexity of care is key.

**Conclusion:**

This review revealed the limited evidence on educational programmes about integrated care for the elderly. Our findings suggest that educational programmes on integrated care should not be limited to the micro-level, and that students should obtain adaptive expertise by experiencing complexity. Future research should contain an explicit description and definition of integrated care.

## Introduction

Following in the wake of healthcare, medical education is itself going through a transition because of the ageing society, which is associated with the rise of chronic diseases and multimorbidity [1,2,3,4,5]. Policy papers on medical education, therefore, indicate what qualities (future) physicians should have to prepare for this transition and emphasise the need for learning integrated care [6,7] without, however, providing information on how integrated care should be taught to (future) physicians.

The elderly currently receive care in a fragmented healthcare system that is primarily designed for providing mono-disciplinary care [3]. Consequently, relevant health issues are not consistently recognised, resulting in high risk of complications, rising healthcare costs, and (frail) elderly not experiencing their healthcare as a cohesive continuum [3,8,9,10,11]. To address this mismatch in the needs of the elderly and the current care provided, integrated care initiatives and evidence are on the rise [9,12,13,14,15]. For the provision of integrated care, healthcare workers need to be skilled to work within the context of integrated care and capable of coordinating complex care [2,3,4]. To ensure greater collaboration within the healthcare sector [16], therefore, programmes were developed that focus on interprofessional learning, i.e. learning from and with different professionals in health and social care. Although interprofessional collaboration contributes to better care integration, it does not encompass the whole concept of integrated care [17]. Interprofessional learning focuses on better collaboration, but other essential components of integrated care such as finance, prevention, and management are not entirely addressed.

Because a single unifying and accepted definition of integrated care is lacking, educational programmes on this topic are not easily found [14]. The concept is multifaceted and can be approached from different perspectives, complicating the search for such programmes [18]. The explanation for this difficulty goes back twenty years to when the concept of integrated care emerged as a counter-reaction to fragmented care [19]. From the perspectives of patients, funders, and healthcare professionals, amongst others, initiatives on integrated care arose, and integrated care, therefore, is subject to different perspectives and terminologies such as ‘comprehensive care’ or ‘coordinated care’ [18,19,20]. In addition, numerous frameworks for integration have been described [12, 14]. Integration can occur at different levels: the micro-level (individual patient), meso-level (organisational/social context), and macro-level (population) of integrated care. Alternatively, integration is possible in different directions, for example, horizontally (e.g. social and health services) or vertically (disease-based) [12, 14,21,22]. This diversity of definitions, perspectives, and frameworks makes research and comparison of integrated care challenging [18,19]. Overviews of integrated care exist for practice. The World Health Organization (WHO), for example, published a document that focuses on integrated care for older people [3], compiled a framework including an elaborated strategy [10], and the WHO Regional Office for Europe has set out an overview of the diverse concepts and models for integrated care [12]. For medical education, such overviews of integrated care do not exist, and this, together with the multifaceted nature of the concept, makes finding and comparing educational programmes quite a challenge.

In order to unite practice, medical education, and the needs of elderly patients, it is necessary to clarify what education on integrated care should entail. Along with practice, education on this topic will develop, and initiatives may already have emerged. It remains unclear whether such educational programmes exist, and if so, on the basis of which theoretical framework they operate and what education these programmes provide. Therefore, this review aims to create an overview of educational programmes, the components they teach, and their outcomes regarding teaching integrated care for elderly patients to undergraduate medical students and postgraduate trainees (physicians pursuing further clinical training to become medical specialists). In this way, this review hopes to contribute to making the necessary transition in medical education, clarify knowledge gaps, and inspire future medical education on integrated care for the elderly.

## Methods

We chose to perform a scoping review as this method is suitable for mapping a broad topic, identifying knowledge gaps, and starting future research [23,24,25]. As a guide, we used ‘The Joanna Briggs Institute (JBI) Manual for Evidence Synthesis’, based on the frameworks of Arksey and O’Malley [24] and Levac et al. [25], and ‘the Preferred Reporting Items for Systematic reviews and Meta-Analyses extension for Scoping Reviews (PRISMA-ScR)’ [23,26]. The scoping review protocol was registered at the Open Science Framework (OSF) [27].

### Search Strategy

With the help of a librarian, we developed a search strategy by dividing the research question into four concepts:

1. Integrated care and its synonyms
2. Elderly and its synonyms
3. Learning and its synonyms
4. Students/trainees, and their synonyms.

For concept 2, we adapted the search string for geriatric people published by van de Glind et al.. To adapt the concept from geriatric to elderly people in general, we replaced disease-specific components, such as ‘Alzheimer’s’, with broader components, such as ‘comorbidity’ and added ages, such as ‘70 years’ [28]. Concepts 3 and 4 were combined with [AND] to avoid papers about patient learning. We combined these concepts in the following Boolean search: (Concept 1 AND Concept 2 AND ((Concept 3 AND 4)). In April 2021, we adapted the final search string to PubMed, Embase, PsycINFO, ERIC, and Web of Science databases (see Appendix 1 for PubMed search string, the remaining search strings can be found in the review protocol at OSF). We searched these databases for English publications, including papers from 2000 onwards because integrated care had been upcoming since the end of the 1990s [20,29,30].

### Selection criteria

The included publications had to describe an *educational programme* for *medical students or postgraduate trainees*, in which they learned to provide or gained insight into *integrated care* for the *elderly*. For this scoping review, we defined the elderly as 65 years and older. We concluded that the health system-based definition of integrated care, documented in the overview of integrated care models by the WHO Regional Office for Europe, best fitted the purposes of our review (**Box 1**) [12]. The first screening showed that none of the full-text papers met the full WHO definition. Therefore, we divided the definition into ten components to determine essential components for this review (**Box 1**).

**Box 1 Health system-based definition of integrated care as used by the World Health Organization (WHO) Regional Office for Europe (2016) [4,12]**

*Integrated health services delivery is defined as an approach to strengthen*

- people-centered* health systems
              *through the promotion of the*
- comprehensive delivery of quality services across the life-course*
- designed according to the multidimensional needs* of the population and the individual
                          *and*
- delivered by a coordinated multidisciplinary team* of providers working
- across settings and levels* of care.
                  *It should be*
- effectively managed* to ensure optimal outcomes
                          *and*
- the appropriate use of resources* based on the best available evidence
- with feedback loops* to continuously improve performance
                          *and to*
- tackle upstream causes* of ill health
                          and to
- promote well-being* through intersectoral and multisectoral actions.

* The words **in bold** are used in the text to refer to the relevant component.

In order not to exclude too many papers based on their abstracts, we decided that at least two out of the ten WHO components should occur during the abstract screening process. During the full-text selection, the review team discussed that all papers should comply with educational programmes targeting component I. (‘people-centred’), component III. (‘multidimensional needs’), component IV. (‘multidisciplinary team’), and component V. (‘across settings and levels’). We decided that the remaining components were desirable but not essential for selection. We chose these four mandatory components because they are within the sphere of influence of (future) physicians. Components such as VIII. feedback loops are more challenging for medical students or trainees to influence as they are not responsible and sometimes not even included in the evaluation of care during their internships.

Interprofessional collaboration, as mentioned earlier, is an important component to ensure greater collaboration within the healthcare sector [16], making IV. multidisciplinary team essential. We defined ‘multidisciplinary team’ as generalists and specialists who collaborate together in health and social care, as mentioned by the WHO in their report as a key factor for implementing integrated care [12]. Currently, for multidisciplinary collaboration, the term interprofessional collaborations (IPC) and intraprofessional collaboration (IntraPC) are also used. IPC means health professionals with different backgrounds collaborating to deliver high-quality care within health and social care [31,32]. IntraPC is defined as health professionals with the same background collaborating to provide care [32]. For this review, we included papers that involved multidisciplinary, interprofessional or intraprofessional collaboration with respect to health and social care.

### Selection

The review team MW, NS, CF, and DA screened 6,936 titles and abstracts using Rayyan [33]. At least two researchers screened every abstract. The abstract screening resulted in 30 papers for full-text reading. During full-text reading, DA and MW scored every paper on the presence of the WHO components (**Box 1**). These WHO components had to be reflected as an intention, learning objective, outcome, or element of the described educational programme. If the researchers intended to develop an educational programme for students to become more patient-centred, for example, we scored that component I. was present.

**Figure 1:**
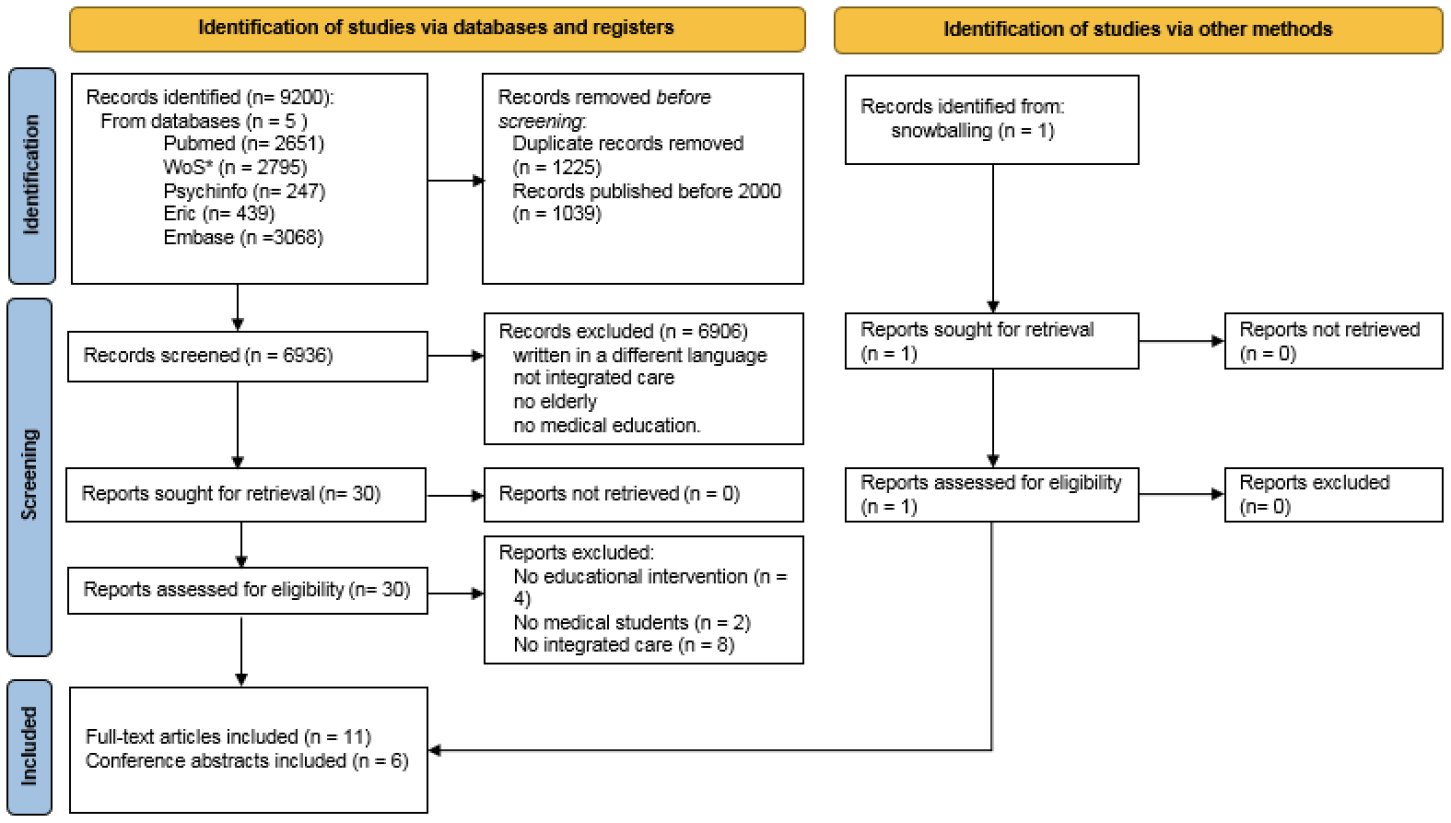
The Prisma flow diagram. * WoS = Web of Science *From*: Page MJ, McKenzie JE, Bossuyt PM, Boutron I, Hoffmann TC, Mulrow CD, et al. The PRISMA 2020 statement: an updated guideline for reporting systematic reviews. BMJ 2021; 372: n71. doi: 10.1136/bmj.n71. For more information, visit: http://www.prisma-statement.org/.

Ten full-text articles and six conference abstracts met the inclusion criteria. The references in these articles were screened in the same way as above, resulting in one additional paper [34]. During inclusion, the review team discussed disagreements. The Prisma flow diagram shows an overview of the selection process (Figure 1).

### Data analysis

All 17 inclusions were read and reread. DA and MW developed and piloted a charting template to collect descriptive and content data. We categorised the data into four main categories:

1. descriptive information (e.g., title, author, country),
2. information regarding integrated care (e.g., definitions, levels, components),
3. information regarding the educational programmes (e.g., relevance, content, setting, educational theories),
4. and information regarding the outcomes of the educational programmes.

During analysis, we defined the micro-level of care as direct (individual) patient care, including the patient’s social system; we defined the meso-level as the organisation of care for (a group of) patients; and we defined the macro-level as the care organisation for the entire population, including government policy, legislation, and finance. The content data were deductively and inductively analysed in Excel and Atlas.ti. using template analysis [35,36]. DA and MW read and reread the data. They analysed, coded and categorised the data independently in Excel and Atlas.ti. They discussed their findings weekly. SG joined the review team during analysis to add expertise on integrated care. The findings and discrepancies were discussed within the review team until consensus was reached. Finally, overarching and several sub-themes were identified, which we describe in the results.

### The review team

The review team consisted of professionals from different fields and backgrounds: a PhD student and a geriatrician in training (MW); a programme director in geriatrics, educator and geriatrician (DA); a strategic consultant and researcher in integrated care (SG); an experienced educationalist and professor of innovative and person-centred learning and working in healthcare (CF); and a general practitioner and professor of general practice, specialising in interprofessional and primary-secondary care collaboration (NS).

## Results

We included 17 papers to address our research objectives. During analysis, we defined the following six themes:

1. description of the educational programmes,
2. content and setting of the educational programmes,
3. educational theories and concepts of the educational programmes,
4. relevance of the educational programmes,
5. components and levels of integrated care addressed by the educational programmes,
6. and outcomes of the educational programmes.

### Description of the educational programmes

The included papers described a wide variety of educational programmes. Table 1 presents an overview of the included papers with participants, brief description, setting, and duration of the educational programmes. Thirteen papers originated from North America [34,37,38,39,40,41,42,43,44,45,46,47,48], two from Europe [49,50], one from South America [51], and one from Oceania [52]. The majority (n = 13) was published after 2008 [37,38,39,41,42,43,44,46,48,50,51,52].

**Table 1:** An overview of the included papers.


FIRST AUTHOR, YEAR OF PUBLICATION, COUNTRY	PARTICIPANTS	DESCRIPTION	CLASSROOM BASED SETTING	WORKPLACE BASED SETTING	DURATION OF THE PROGRAM

E.S. Anderson, 2010, United Kingdom	Medical, nursing, social work, and speech and language therapy students. They are all completing practice learning placements towards the end of their training.	The students cared for one in-patient, explored discharge processes and policies, and considered how the social and medical models of care are combined to support patient choice based on their needs. They explored the contributions of all members of the ward clinical team. At the end of the week, the student team presented their patient case to the ward team offering solutions to problems in an interactive feedback session.		•	4 or 5 days

S.A. Balogun, 2015, United States	Third-year medical and fourth-year nursing students.	The workshop features a clinical case of a woman with dementia that is being transitioned from the hospital to her home. The workshop addresses interprofessional communication and issues on discharge from the hospital and at home.	•		90 minutes

S.E. Hart, 2021, United States	Interprofessional teams of four to six students, representing medicine, nursing, pharmacy, public health, physical therapy, and social work.	Students finish a foundational curriculum after which teams of students from different professions are paired with patients having complex needs (student hotspotting).	•	•	2 hours/week for 6 months

M. T. Heflin, 2013, United States	Two students from each profession with a target total of six to 12 students in medicine, nursing, physical therapy, physician assistants, pharmacy, and social work.	To learn to improve transitions in care, interprofessional teams work on quality improvement projects: a series of learning experiences consisting of in-person sessions, between course readings and practical exercises, and web-based discussions.	•		The fall semester

T. Imam, 2019, United Kingdom	Geriatric specialist trainees and GP registrars.	Joint GP-geriatric trainee clinic within primary care		•	6 months

F. Kent, 2014, Australia	Fourth- or fifth-year medical students and final- year students from nursing, nutrition and dietetics, occupational therapy, physiotherapy, podiatry, pharmacy, psychology, social work and speech pathology	Interprofessional student-led-aged clinic for recently discharged elderly where they take care of their unmet healthcare needs		•	Not specified

M.E. Keough, 2002, United States	Medical residents and family practice residents.	On-site interactive seminars focused on effective communication based on a clinical case selected by team members of the Elder Service Plan group (ESP). The ESP program is an all-inclusive care program to preserve the health and independence of its participants.	•		Not specified

A. Lathia, 2015, United States	Medical students within the geriatrics rotation at the Cleveland Clinic.	A mixed program of discussions – tour through the care units – observations – talking about in-service problems. The program supplements standard geriatric didactics during the medical student primary care rotation about transitions of care.	•	•	7 hours

Y.S. Meah, 2012, United States	Third year medical students.	Non-traditional longitudinal interdisciplinary clerkship (LIC): foundational ambulatory care venues of the standard curriculum traditionally taught singularly during the block clerkships are transformed into a multidisciplinary integrated longitudinal experience.		•	8-11 weeks

M.C. Mecca, 2014, United States	An interdisciplinary group of trainees within the Veteran Affairs Connecticut Center for Excellence in Primary Care Education.	Students are offered a multifaceted curriculum that includes interactive didactic sessions co-led by a geriatrician and allied health service staff, in addition to clinical experiences for translating education into practice. If they are interested, students are offered to perform a quality improvement project.	•	•	Not specified

L. B. da Motta, 2014, Brazil	Residents (defined as a graduation course) in medicine, nursing, physiotherapy, nutrition, psychology, and social services.	Six longitudinal interprofessional practical scenarios: outpatient, infirmary, educational actions, neurogeriatric, home based care, and long term institutionalisation. They are taught over two years within the residency program and increase complexity.	•		2 years

K. Ouchida, 2009, United States	Third-year medical students completing their required internal medicine rotations.	An education intervention to foster essential elements of transitional care by Fast Forward Rounds: an interactive education program with lectures, interactive video, small-group discussion, and a team-based learning exercise.	•		2 x 90 minutes

S. Saffel-Shrier, 2012, United States	Second- and third-year family residents.	Family residents provide the primary care of two patients who live within an assisted living facility. The interprofessional faculty team supervises them.		•	2 years

J. Thornhill, 2002, United States	Medical students (undergraduate).	Students are paired with healthy elderly. During four years, students follow these elderly, talk about their multidimensional needs, and consult other healthcare professionals. The modules were designed to coordinate with other areas of the curriculum so that the students have an opportunity to put the learned concepts into practice.	•	•	4 years

D. Vincent, 2014, United States	Medical residents	Follow-up home visits by a multidisciplinary team led by residents after the patient was discharged from the hospital.		•	Not specified

G.C. Xakellis Jr, 2003, United States	Students in nursing, social work, public health, health administration, and medicine	A web tool that allows the learner to grapple with the essential challenges of improving care provided to a Medicare-aged population in the current health care environment. Students were asked to manage the health of a population of 5,000 seniors by dividing them into three categories: basically healthy, moderately ill, and severely ill.	•		It can be used as the basis for a one-time class discussion, a multiweek group project, or a complete master’s thesis.

S. Yang, 2019, Canada	Medical students	Students role-played an elderly patient (with complex health needs) or their caregiver within five simulated healthcare professional appointments.	•		150 minutes


Thirteen papers contained educational programmes for undergraduate students [34,37,38,39,41,42,45,46,47,48,49,51,52] and four for postgraduate trainees [40,43,44,50]. The duration of the programmes varied between 90 minutes and four years (Table 1). The duration was not specified in four papers [40,43,46,52].

### Content and setting of the educational programmes

We subsequently divided the programmes according to their learning settings, i.e. classroom-based, workplace-based, or a mix of both (Table 1). Six programmes had a classroom-based setting [34,37,39,40,47,48]. These programmes varied from interactive seminars based on a clinical case [34,40], a workshop based on a clinical case [37], an application of a web-based tool to manage care on a population level [47], a quality improvement project [39], and a scenario play [48].

The remaining 11 papers described workplace-based programmes. One described a non-traditional longitudinal interdisciplinary clerkship (LIC) incorporating ambulatory care within longitudinal education [42]. Another complemented regular resident rotations with six longitudinal interprofessional curriculum components taught over two years [51]. Other activities included home visits [43,46], joint primary and secondary care clinics [50], ward observations [41], a student-led clinic for recently discharged elderly [52], and an interprofessional student team examining one patient’s care and discharge [49]. Lastly, these papers described students or residents paired with elderly persons to provide care [44] or observe their health needs [38,45].

Four of these workplace-based programmes also used teaching activities in a classroom-based setting [38,41,43,51]. For example, students completed a foundational curriculum, after which they continued with home visits to patients [38].

#### Multidisciplinary setting

All the educational programmes addressed component IV. (‘multidisciplinary team’). In eight programmes, the participants participated in teams of students from different professions in healthcare [37,38,39,47,49,51,52,53] of which seven programmes included students from social work [38,39,47,49,51,52,53]. In two papers, participants participated in a multidisciplinary team that was not made up exclusively of learners but also included working professionals in health and social care [40,46]. In three programmes, students received supervision or learned from an interprofessional team of healthcare professionals [34,44,45]. One programme had an intraprofessional learning setting [50], and in one programme, participants received supervision from different medical specialists [42]. Another paper described an interactive session with other healthcare professionals [41], and the last programme described a simulation of a patient who visits multiple health and social care workers [48]. Three programmes featured social care without interaction with a social care worker [34,37,48], and five programmes did not mention social care [41,42,44,45,50].

### Educational theories and concepts

Five papers mentioned an educational or theoretical concept on which their programme was based. One paper described the concept of situated learning, in which learning requires interaction and collaboration [52]. The second paper described a productive struggle to ensure students’ understanding of the complexity of care experienced by patients [48]. The third paper mentioned the pedagogical guideline of problem-solving, in which the students were encouraged to reflect on their actions concerning the actions of other professionals [51]. Another paper described the Leicester Model of Interprofessional Education, which taught students to value teamwork [49]. Finally, the last paper based their programme on problem-based learning, which stimulates creative solution finding and teaches students how to cope with complex problems on a population level [47].

### The relevance for developing the educational programme

Thirteen papers mentioned the relevance of developing education on providing care for the elderly. The relevance items mentioned were the ageing society, the increasing complexity of care demands, and the shortage of geriatricians [34,37,38,39,40,41, 43,44,45,47,48,51,52]. Four papers, however, did not motivate why they taught (a component of) integrated care [42,46,49,50].

### Components of integrated care

Based on the inclusion criteria, all programmes contained the following components (**Box 1**):

- people-centred
- multidimensional needs
- multidisciplinary team
- across settings and levels.

Although we searched for elements of the concept of integrated care, only two papers mentioned integrated care as a learning objective, but they did not define the concept of integrated care [48,50]. Table 2 provides an overview of the components of integrated care in the WHO definition that the educational programmes taught. Sixteen programmes included additional components to the required four [34,37,38,39,40,41,42,44,45,46,47,48,49,50,51,52]. Component VI. (‘effectively managed’) was taught in 13 programmes, making it the most widely taught additional component [34,37,38,39,40,41,44,46,47,48,49,50,51]. This was followed by component II. (‘across the life-course’) [34,42,45,47,51,52] and IX. (‘tackle upstream causes’) [34,40,45,47,51,52], which were both taught in six programmes. Two programmes taught Component VII. (‘feedback loops’), which was, therefore, the least taught component [39,47].

**Table 2:** Overview of the taught components of the WHO definition.


FIRST AUTHOR, YEAR OF PUBLICATION	I. PEOPLE- CENTERED HEALTH SYSTEMS	II. COMPREHENSIVE DELIVERY OF QUALITY SERVICES ACROSS THE LIFE-COURSE	III. DESIGNED ACCORDING TO THE MULTIDIMENSIONAL NEEDS OF THE POPULATION AND THE INDIVIDUAL	IV. DELIVERED BY A COORDINATED MULTI- DISCIPLINARY TEAM OF PROVIDERS WORKING	V. ACROSS SETTINGS AND LEVELS OF CARE	VI.EFFECTIVELY MANAGED TO ENSURE OPTIMAL OUTCOMES	VII. THE APPROPRIATE USE OF RESOURCES BASED ON THE BESTAVAILABLEEVIDENCE	VIII. WITH FEEDBACK LOOPS TO CONTINUOUSLYIMPROVE PERFORMANCE	IX. TACKLE UPSTREAM CAUSES OF ILL HEALTH	X. PROMOTE WELL-BEING THROUGHINTERSECTORAL ANDMULTISECTORAL ACTIONS

E.S. Anderson, 2010	•		•	•	•	•		•		

S.A. Balogun, 2015	•		•	•	•	•				

S.E. Hart, 2021	•		•	•	•	•				

M. T. Heflin, 2013	•		•	•	•	•	•	•		

T. Imam, 2019	•		•	•	•	•				

F Kent, 2014	•	•	•	•	•				•	•

M.E. Keough, 2002	•		•	•	•	•			•	

A. Lathia, 2015	•		•	•	•	•				

Y.S. Meah, 2012	•	•	•	•	•					

M.C. Mecca, 2014	•		•	•	•					

L. B. da Motta, 2014	•	•	•	•	•	•		•	•	

K. Ouchida, 2009	•	•	•	•	•	•			•	•

S. Saffel- Shrier, 2012	•		•	•	•	•				

J. Thornhill, 2002	•	•	•	•	•				•	

D. Vincent, 2014	•		•	•	•	•				

G.C. Xakellis Jr, 2003	•	•	•	•	•	•	•		•	•

S. Yang, 2019	•		•	•	•	•				•


#### Levels of integrated care

As explained previously, integrated care can be considered at micro-, meso- and macro-levels. Except for the paper by Xakellis and Robinson, all programmes focused on the micro-level of integrated care [34,37,38,39,40,41,42,43,44,45,46,48,49,50,51,52]. Within the programmes focusing on the micro-level, four programmes incorporated the meso-level [34,44,48,49], four the macro-level [39,40,41,51], and two incorporated the meso- and the macro-levels [37,38]. For example, Balogun et al. taught students about the collaboration and financing of different healthcare organisations in the preparation of the workshop [37]. The programme described by Xakellis and Robinson is unique as its focus is primarily on the macro-level, with students being challanged to allocate disease prevention funds to average and seriously ill elderly. The students also learned to think on a meso-level by devising an organisational strategy [47].

### Outcomes

The authors evaluated their programmes to a greater or lesser extent. Some papers did not specify their evaluation methods [42,45,47], while others provided a preliminary evaluation [40], and evaluations varied from detailed to short descriptions in the remaining papers [34,37,38,39,41,43,44,46,48,49,50,51,52]. For clarity, we divided the heterogenous evaluation outcomes into the following categories: feedback on the programme, learning outcomes, key factors for learning, and challenges.

#### Feedback on the programme

Twelve papers provided feedback on the programme. Two papers provided patient feedback [44,50]. The remaining papers mainly provided student feedback [34,37,39,41,42,43,45,46,49,52]. Eight papers were limited to student feedback [34,37,39,41,42,43,45,46], two papers also provided educator feedback [46,49], one paper provided student and patient feedback [45], and one paper included student, educator, and patient feedback [52].

Students were positive about the longitudinal mentorship and their progress in skills [42], discussions with patients about care settings [41], interactive formats [34,39], the flexibility of self-directed learning [49], clinical experience [39,45,46], contacts with patients and family caregivers in a non-regulated environment without time pressure [46], quality improvement projects, and the instructor preparation for the programme [39]. Students indicated that clarification of the levels of care was helpful, for example, with a pocket card [41]. Students suggested that the programme could be improved in terms of the level of involvement of other professions [37,52], the timing [34], and the effectiveness [52].

Educators were positive [46,52] and appreciated students’ input on the ward [49]. One educator, for example, was pleased because the students had prevented a discharge from failing [49]. For improvement, educators would like to be better trained in providing interprofessional education [52].

Patients were also positive [45,50], appreciating, for example, the interaction with students [45] and the referrals by students to other health services [44,52].

#### Learning outcomes

The learning outcomes varied. Firstly, there were learning outcomes focusing on the WHO components of integrated care (Table 2). In addition, surveys showed enhanced student attitudes and self-efficacy in caring for the elderly in different settings [39,41]. Students reported more awareness of the impact of policy on daily practice [49], improved communication [37], and an enhanced understanding of social and environmental aspects of healthcare [46]. Xakellis and Robinson hypothesised that the participating students experienced the clinical and financial sides of healthcare and indicated that the small sample of patients in their teaching could have contributed to providing an accessible way of applying care strategies at the population level to improve individual patient care [47]. Qualitative analyses from Yang et al. showed that students experienced the complexity of care [48]. Lastly, students indicated they had to use their sense of responsibility [42,49,51] and reported a willingness to focus more on the patient’s perspective [34,37,39,48,49].

#### Key factors for learning

Ten papers suggested factors that contributed to the students’ learning process [34,37,41,42,46,48,49,50,52]. Three papers described that observing and experiencing care outside the hospital helped to clarify the different levels of care [41], prevent deterioration [46], and take responsibility [42]. Experiencing care through simulation of [34,37,48] or interaction with patients [49,52] helped students to gain insight into the patients’ perspective, among other things. Participating in joint clinics contributed to better collaboration between the different levels of care [50]. Da Motta and Pacheco added that by offering complexity of elderly care to a team of students from different disciplines, students had to appeal to each other’s expertise, which taught them to work together in a cooperative way [51]. Yang et al. suggested that complexity as part of their scenario play allowed students to encounter the patients’ experience. The struggle with the patients’ care navigation provided a better understanding of integrated care, which stimulated the students’ critical self-reflection, allowing them to better empathise with patients [48]. These results suggest that complexity as an educational element and experiencing patients’ care navigation contributed to the students’ learning process.

#### Challenges

Several challenges in the implementation of these programmes were mentioned [34,37,41,45,49,50,51,52]: sufficient financial resources [38,52], different levels of experience among learners [37,39], enough time for supervision [41,49], enough time or flexibility within the curriculum [41,45,50], finding qualified supervisors [42,52], the students’ assessment [42], the right location [41,42,49,52], and receiving support for the educational programme from hospitals or educational institutions [49,51,52].

## Discussion

To the best of our knowledge, this scoping review is the first to outline published papers on teaching integrated care for the elderly and its challenges and key factors for learning. The 17 selected papers described various educational programmes in classroom-based, workplace-based or a mix of both settings and had various educational theories or concepts as their foundation. None of the papers mentioned a specific definition of integrated care.

All articles described similar challenges for developing their education programme, namely the ageing society and the increasing complexity of care. Integrated care is put forward as a possible solution to these challenges. Considering the definition we used, i.e. the WHO definition for integrated care (**Box 1**), we noticed that – spread over the papers – all ten components were reflected in the educational programmes to a greater or lesser extent. It is noticeable that, in addition to the four components used for inclusion, only VI. (‘effectively managed care’), was frequently addressed. The five remaining components of the WHO definition, in contrast, appeared in less than half the programmes. The included programmes did not have integrated care as such as their starting-point, but had the challenges and complexities of the ageing society as their foundation, which may be the reason why a definition of integrated care was not used. Based on our research, we cannot clarify why certain components of the WHO definition recurred less frequently.

As described in the introduction, learning to participate in a multidisciplinary team is essential in integrated care. Also, a previous literature review on educational needs for educating nurses in medical education suggests that interprofessional learning is a crucial component in learning integrated care [54]. Therefore, component IV. (‘multidisciplinary team’) was mandatory for inclusion. Our results showed that students emphasized the need for interprofessional collaboration [37,52], and that interprofessional learning improved collaboration [50,51]. The educational programmes found implemented component IV. (‘multidisciplinary’) team differently. Two programmes addressed **intra**professional learning [42,50], and the remaining programmes included **inter**professional learning [34,37,38,39,40,41,44,45,46,47,48,49,51,52,53]. Five programmes did not address social care [41,42,44,45,50], which is understandable in the case of Imam, T., et al. and Meah, Y. S., et al. given that these programmes were set up as intraprofessional learning programmes within the medical profession [42,50]. The connection with social care is important within interprofessional education [31]. Therefore, it is notable that three interprofessional learning programmes did not address social care [41,44,45], and three other programmes only mentioned social care without involving social caregivers [34,37,48]. Also, when we look at research about social care within integrated care and the definition of integrated care (i.e. component III. (‘multidimensional needs’), social care is important in integrated care [12, 55]. Therefore, we recommend that educational programmes towards integrated care should pay attention to multidisciplinary collaboration, including social care.

In addition to the WHO components, we examined the different levels of integrated care that were taught. The main focus of the programmes was the micro-level of integrated care with extensions to the other two levels. This finding is consistent with the integrated care approaches for the elderly in practice, which focus mainly on the micro-level [56]. Nevertheless, 11 of the 17 educational programmes did introduce students to the other levels of integrated care by, for example, embedding education on the organisation of care (meso-level) or population-level prevention (macro-level) into their programme. Whether this is an essential element for learning about integrated care remains unclear. However, previous studies show that it is important for students to become acquainted with levels other than the micro-level in their education. For example, the ‘framework of integrated competencies for adaptive expertise on integrated care’ [57] describes that it is essential to know the patient and his or her care system and social system. Westerman et al. also emphasised that postgraduate medical education should address both the delivery of patient care and other factors, such as the finance and management side of healthcare [58]. In a study on education about integrated care for trainee paediatricians, in addition, trainees indicated that they felt it was important to learn about the whole of integrated care and learn to adapt to changes at the macro level [59]. Therefore, we suggest that education should not be limited to the micro-level of care.

Furthermore, our data imply that first-hand participation and experience contributed to learning how to deliver integrated care [34,37,41,42,46,48,49,50,51,52]. These experiences should not be oversimplified [34,37,48,49,52]. The students’ struggle with the reality of complex care for the elderly made them willing to provide patient-centred care and increased their understanding of integrated care. These programmes had in common that students experienced the complexity of care by putting themselves in the patients’ position (e.g., through simulation) or were made responsible for the patients’ comprehensive care. In addition, providing students with the authentic complexity of contemporary care contributed to learning to work cooperatively [51]. Rather than a simplified, so-called reductionist approach, complexity science reflects an approach in which the patient is considered as a complex system [60,61]. In this scientific movement, health care consists of different agents who interact in so-called Complex Adaptive Systems (CAS) [60]. These CAS succeed because their agents, rather than attempt to find a fixed solution, can adapt to changes in the system. This requires that students should gain adaptive expertise [57,62,63]. Sockalingam et al. argued that adaptive expertise could be meaningful in providing integrated care. They suggest that students would ideally first learn the scientific basis of diseases and their treatment before experiencing in practice or simulation how multimorbidity, the patient’s context, and the doctor’s preferences interact. This way, students would get the opportunity to experience care, struggle with it, and learn to adjust it in an integrated way, and thus learn about providing integrated care [63]. This aligns with a scoping review on educational programmes for collaborative care in psychiatry, in which the authors conclude that adaptive expertise is important to prepare students for addressing complexity [64]. Therefore, it seems essential for students to experience the complexity of healthcare. In this way, students could acquire the skills needed to deliver integrated care in the future.

### Strengths & Limitations

We would like to mention two strengths. Firstly, our diverse research team, consisting of educators, physicians, and a policy and management expert, allowed us to analyse the included papers from different perspectives. Secondly, we operationalised the WHO definitions of integrated care into ten components (**Box 1**), which made it possible to perform a broad search and identify the components of integrated care that the educational programmes addressed. Nevertheless, only 17 papers were electable, a limited number of papers that contrasts rather sharply with the global trend towards more integrated care. A lack of research publications does not necessarily mean that there is no education on integrated care. Nevertheless, this scoping review has exposed the limited extent of scientific knowledge and highlights the need for research regarding educational programmes on integrated care for the elderly.

A limitation is perhaps that we did not include grey literature. Because of the broadness of the concept of integrated care, searching with the same broad scope as in white literature would have led to an unworkable amount of information. A pilot search in grey literature only revealed programmes on integrated care in general, which did not address the elderly. Therefore, grey literature remained outside the scope of this review.

Another limitation could be the quality of the included papers. Some papers were very brief in describing their programmes and evaluations. Some evaluations were limited to stating that ‘students enjoyed the programme’ without clarifying what it was they enjoyed. Not all papers described the relevance of their educational programme, which made it challenging to understand the setting of the programme. The generalisability of the data, therefore, is limited. However, we believe that this review can be used as a source of inspiration and an incentive for developing education and research.

### Implications for medical education

The described educational programmes did not have integrated care as a primary learning objective but taught components of the WHO definition of integrated care. Therefore, this review does not provide evidence on the effectiveness of educational interventions on the full spectrum of integrated care. Nevertheless, this research shines a light on interventions for components of integrated care and can be used as an inspiration for developing education on this subject. In addition, we suggest promoting a broad scope in medical education on integrated care, by addressing, firstly, the broader meso-level and macro-level of integrated care and, secondly, by enabling students to experience the complexity of care. Furthermore, we suggest considering the challenges described: sufficient financial resources, location, the difference in students’ levels of experience, teacher qualifications, enough time, and support from hospitals and educational institutions.

### Implication for future research

Future research should focus on descriptions of educational programmes that pay specific attention to a definition of integrated care to justify the choices made. It may be valuable to include information from educational programmes on integrated care that were outside the scope of this review, such as paediatrics or psychiatry. In addition, future research could examine what components of integrated care the educational programmes should address and why. Furthermore, most programmes focused on the micro-level of integrated care, and it remains unclear how the meso- and macro-levels of integrated care could be included. It would be a valuable addition, therefore, to investigate how these levels could be included in medical education.

## Conclusion

Despite the global trend towards integrated care, none of the included articles aimed for integrated care as the main objective of their educational programme. They addressed only components of integrated care. With this scoping review, we exposed the limited knowledge that is currently available and highlighted, moreover, the need for more research on educational programmes about integrated care for the elderly. The results suggest that exposing students to the various complexities and levels of integrated care could contribute to learning about providing integrated care. We also recommend educational programmes towards integrated care should pay attention to multidisciplinary collaboration, including social care. However, more research about integrated care in healthcare education is necessary.

## Figures and Tables

**Appendix 1:**
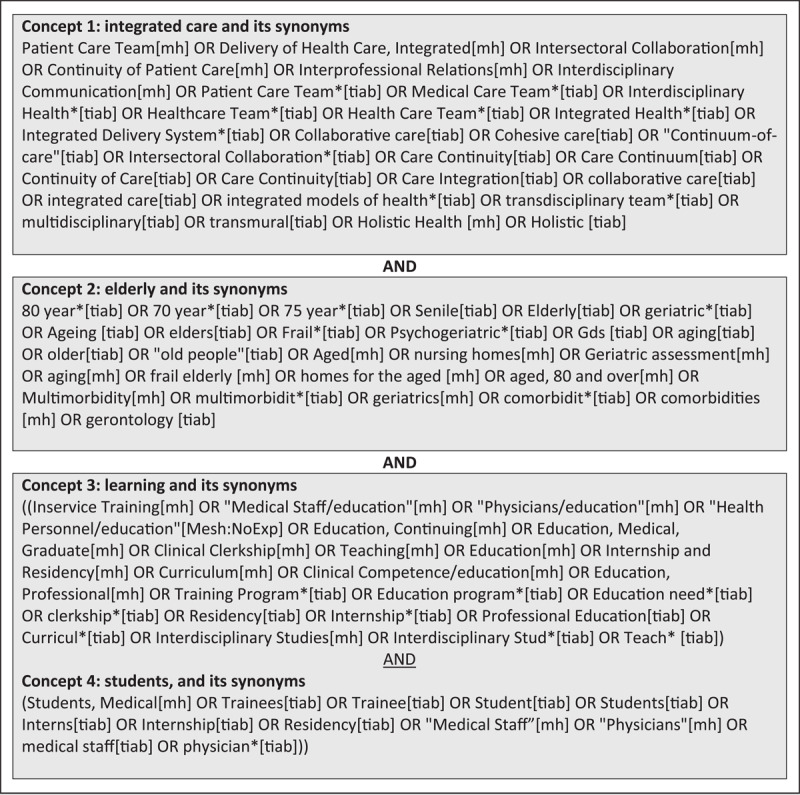
Example of PubMed search string.
